# Use of *Guazuma ulmifolia* Lam. Stem Bark Extracts to Prevent High-Fat Diet Induced Metabolic Disorders in Mice

**DOI:** 10.3390/ijms25168889

**Published:** 2024-08-15

**Authors:** Elisana Lima Rodrigues, Lidiani Figueiredo Santana, Valter Aragão do Nascimento, Marcel Asato Arakaki, Claudia Andréa Lima Cardoso, Wander Fernando de Oliveira Filiú, Rita de Cássia Avellaneda Guimarães, Priscila Aiko Hiane, Karine de Cássia Freitas

**Affiliations:** 1 Graduate Program in Health and Development in the Central-West Region of Brazil, Federal University of Mato Grosso do Sul-UFMS, Campo Grande 79070-900, MS, Brazil; elisana.lima10@gmail.com (E.L.R.); valter.aragao@ufms.br (V.A.d.N.); rita.guimaraes@ufms.br (R.d.C.A.G.); priscila.hiane@ufms.br (P.A.H.); karine.freitas@ufms.br (K.d.C.F.); 2 Course of Medicine, State University of Mato Grosso do Sul, Campo Grande 79804-970, MS, Brazil; 3 Medical School, Federal University of Mato Grosso do Sul, Campo Grande 79070-900, MS, Brazil; marcel_arakakiasato@hotmail.com; 4 Course of Chemistry, State University of Mato Grosso do Sul, Dourados 79070-900, MS, Brazil; claudia@uems.br; 5 Faculty of Pharmaceutical Sciences, Food and Nutrition, Federal University of Mato Grosso do Sul (UFMS), Campo Grande 79079-900, MS, Brazil; wander.filiu@gmail.com

**Keywords:** antioxidants, high-fat diet, medicinal plants, phenolic compounds

## Abstract

This study aimed to evaluate the effects of supplementation with ethanolic and aqueous extracts from the bark of the stem of *Guazuma ulmifolia* in mice submitted to a high-fat diet as well as to evaluate the chemical composition of these extracts. The chemical composition and antioxidant potential was evaluated in aqueous and ethanolic extracts of the stem bark. The in vivo test consisted of evaluating the effects of the aqueous and ethanolic extracts of the stem bark on C57BL/6 mice receiving a high-fat diet. The animals were evaluated for weight gain, feed consumption, visceral adiposity, serum, and inflammatory and hormonal parameters. The results of the chemical analyses corroborate those obtained by the literature, which reported gallocatechin, epigallocatechin and epigallocatechin gallate. Compared with the ethanolic extract, the aqueous extract showed greater antioxidant capacity. Both extracts resulted in lower feed consumption in the animals, but they did not influence weight gain or visceral adiposity and resulted in varied changes in the lipid profile. In addition, they did not influence glucose tolerance, insulin sensitivity, or fasting blood glucose. Furthermore, the leptin levels increased, which may have contributed to satiety, but this was shown to have a negative impact on other inflammatory and hormonal parameters. Therefore, under the conditions of this study, the biologically active compounds present in the plant species *Guazuma ulmifolia* were not able to contribute to the treatment of metabolic changes related to the consumption of a high-fat diet.

## 1. Introduction

The consumption of high-calorie and high-fat diets, especially those that are high in saturated fats, results in an excessive increase in adipose tissue, especially in the abdominal region [1], which is decisive for the development of obesity and other metabolic disorders, such as high blood pressure, dyslipidemia, insulin resistance, diabetes mellitus, and metabolic syndrome [2].

Adipose tissue is considered the most important energy storage organ in the human body, with excess energy that is consumed being converted into triacylglycerol molecules under the action of the hormone insulin; in a situation of energy restriction, energy stores are quickly mobilized under the influence of catecholamines and other lipolytic hormones [3,4]. However, since the identification of leptin, a hormone secreted by adipocytes, whose effect on the central nervous system and endocrine function confers active participation in the control of energy expenditure and appetite, functions have been added to adipose tissue, giving it the role of a multifunctional organ, producer, and secreter of numerous bioactive peptides and proteins called adipocytokines. This concept attributes an important endocrine function to adipose tissue—that of maintaining intense communication with other organs and organic systems [5].

Hormones produced by adipose tissue influence a variety of physiological processes, including the control of food intake, energy homeostasis, insulin sensitivity, angiogenesis, vascular protection, pressure regulation, and blood clotting. Thus, it appears that changes in the secretion of adipocyte hormones resulting from the hypertrophy and/or hyperplasia of adipocytes may constitute a situation related to the genesis of the pathophysiological process of obesity and other metabolic complications [3].

In the search for alternatives for the treatment of metabolic complications, the use of plants for medicinal purposes is an ancient practice of humanity, and their use has demonstrated high potential for healing, maintaining health, and preventing and improving illnesses or their aggravation through clinical, scientific, and experimental findings. It should be noted that the pharmacological validation of medicinal plants that are already in popular use can greatly benefit people with a low income, as they have reduced costs [6].

A species popularly used to treat various diseases is *Guazuma ulmifolia* Lam. (Malvaceae) [7], which is commonly known as “mutamba” [8] or “guácimo” [9]. It is found in Latin American countries, including Brazil, and it occurs especially in Cerrado formations [10]. The botanical parts generally used in folk medicine are the stem bark and leaves of *G. ulmifolia*, and they are generally used in the form of infusions or decoctions [11]. Evidence has confirmed the antidiabetic effect of the stem bark and leaves of *G. ulmifolia* [12,13] and revealed the hypotensive and vasorelaxant effects of the stem bark [14]; hypocholesterolemic effects of the plant have also been reported [15].

Phytochemical studies conducted with the bark of the stem revealed the presence of several bioactive substances, which include phenolic compounds, namely, proanthocyanidins (condensed tannins), glycosylated flavonoids, and aglycones [14,16,17], as the main molecules. These compounds have been reported in the literature to have antioxidant activity [18,19], and this may contribute to the mentioned pharmacological activity.

In this context, the objective was to evaluate the chemical composition of the extracts as well as to evaluate the effects of supplementation with ethanolic and aqueous extracts from the bark of the stem of *G. ulmifolia* in different doses in C57BL/6 mice on a high-fat diet.

## 2. Results

### 2.1. Phytochemical Composition, Antioxidant Activity, and Identified Compounds

The classes of secondary metabolites in the ethanolic and aqueous extracts of the stem bark of *G. ulmifolia* were identified according to the intensity of the characterization reaction; thus, the main compounds found were tannins with a medium reaction intensity (75%) and phenolic compounds and flavonoids with low reaction intensity (50%). In addition to these, coumarins, triterpenes and steroids, cyanogenic heterosides, cardioactive heterosides, and saponins were also identified with lower reaction intensity (Table 1). Comparing the extracts, the aqueous extract had a higher tannin content, while the ethanolic extract had a higher content of phenolic compounds. 

The results of the antioxidant activity of the extracts, which are presented in Table 1, are expressed according to the IC_50_ value, which represents the percentage of the sample necessary to reduce the initial concentration of DPPH by 50%. The lower this value, the greater the antioxidant capacity of the substance. 

The IC_50_ values for the aqueous and ethanolic extracts of *G. ulmifolia* were 37.3 μgmL^−1^ and 45.2 μgmL^−1^, respectively, with the aqueous extraction demonstrating greater antioxidant capacity compared to the ethanolic extract, as it presented a lower IC_50_ value.

Furthermore, in the aqueous and ethanolic extracts of the stem bark of *G. ulmifolia*, nine compounds were identified, and they are presented in Table 2. Among the identified compounds, gallocatechin, epigallocatechin, and epigallocatechin gallate were present in greater quantities in both extracts.

### 2.2. Experimental Protocol

#### 2.2.1. Acute Toxicity Test

**Figure 1:**
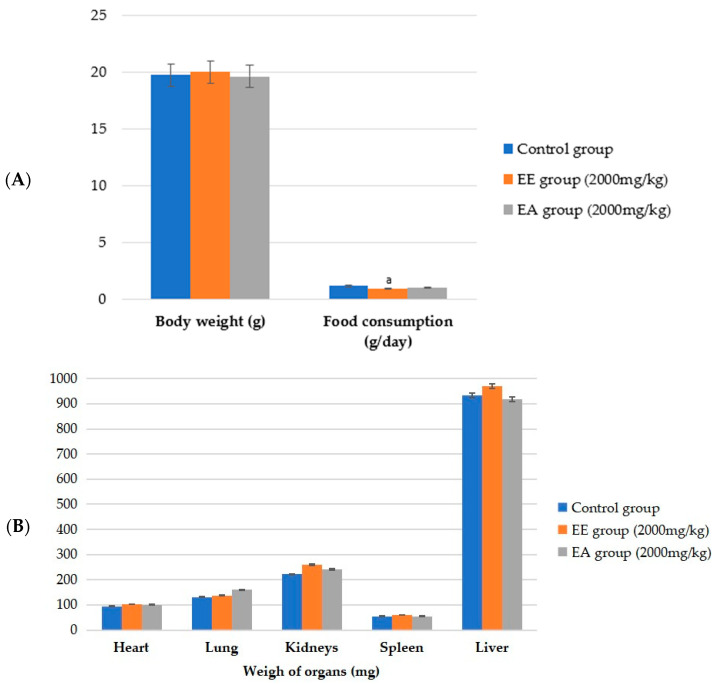
Values of animal weight, food consumption, and weight of vital organs of C57BL/6 mice (females) in the acute toxicity test. (**A**) Weight of vital organs; (**B**) body weight and food consumption. EE = ethanolic extract from the bark of the stem of *G. ulmifolia*. EA = aqueous extract of the stem bark of *G. ulmifolia*. Values are expressed as the mean ± standard deviation. ANOVA followed by the Tukey and Kruskal–Wallis post hoc tests and by Dunn’s test (*p* ≤ 0.001). The letters indicate statistically significant differences as follows: a indicates a difference with respect to the control.

The results indicated that there were no signs of systemic toxicity with no significant differences in body weight among the control, ethanolic extract, and aqueous extract groups (Figure 1A); in addition, there were no changes in water intake or urine and feces excretion. No changes that were motor and/or sensory or neurological in the Hippocratic screening test were observed [20], nor were there any deaths of any animals over the 14-day observation period. The group that received the ethanolic extract had a statistically significantly lower food consumption (*p* ≤ 0.001) when compared to the control group. This effect was expected because one of the objectives was to reduce body weight through the lower food intake resulting from the use of plant extracts. After euthanasia, no macroscopic changes were found in the liver, spleen, lungs, heart, and kidneys of the animals, and there was no significant difference in the weight of the organs between the groups (Figure 1B). Therefore, in this study, the LD50 (50% lethal dose—dose that killed 50% of the animals) of the ethanolic and aqueous extracts of the stem bark of *G. ulmifolia* was greater than 2000 mg/kg of the body weight.

#### 2.2.2. Control of Body Weight and Food Intake

Regarding the body weight results, it is noted that the experiment began with no difference between the control groups (DN, DC, and DH) and the experimental groups (EE25, EE50, EA25, and EA50) in terms of the average weight of the animals. At the end of the experiment, it was observed that the groups remained without statistical differences between them (*p* = 0.480). However, the EE50 group had a higher average weight that was close to that of the DH group. For the total weight gain, calculated as the difference between the final and initial weight of the animals, the EE50 group showed a statistical difference (*p* = 0.032) with greater weight gain compared to the DC control group (Table 3).

With regard to the total food intake (Table 3), it was possible to observe that the EE25 and EA50 groups had a lower average consumption compared to all control groups, and the difference was statistically significant (*p* ≤ 0.001). The EE50 and EA25 groups differed significantly from the DN and DC groups, maintaining a lower food intake.

In relation to daily caloric intake, it is worth highlighting that the EE25 and EA50 groups presented lower values with a significant difference when compared with the DH group (*p* ≤ 0.001). When evaluating the CEA parameter, the EE50 group differed from the DC and DN groups by presenting higher values, and the EA25 and DH groups only differed from the DC group, also with higher values. For the CGPCC parameter, the DC group presented a lower value than did the DN, EE50, EA25, and EA50 groups, with a statistically significant difference (Table 3). Thus, it was observed that under the conditions of this study, there was lower food consumption in the group that received a lower dose of the ethanolic extract and a higher dose of the aqueous extract; there was a difference from the control groups, but there was no influence of both extracts on the gain of body weight in the animals during the 8 weeks of follow-up.

#### 2.2.3. Assessment of Body Fat

When evaluating the weight (g) of the epididymal and retroperitoneal adipose tissue sites, the DC group had a significantly lower weight when compared to the EE25, EE50, EA25, EA50, and DH groups (*p* < 0.001). Furthermore, the DN group significantly differed from the EE25, EE50, and EA25 groups, maintaining a lower weight of the epididymal and retroperitoneal sites (Table 4).

In the perirenal tissue, the DC group obtained significantly lower values compared to the DH, EE25, EE50, and EA25 groups (*p* < 0.001). In the mesenteric and omental adipose tissues, there were no significant differences between the groups. The DC control group had the lowest percentage of adiposity with a significant difference from all groups that received a high-fat diet (*p* ≤ 0.001).

Regarding the area of adipocytes in the epididymal adipose tissue, we observed that there was a significant increase in the DH and EA25 groups when compared with the DN and DC control groups, as shown in Table 4. The EE25, EE50, and EA50 groups significantly differed only from the DC control group (*p* ≤ 0.001).

### 2.3. Glycemic Profile: Oral Glucose Tolerance Test, Insulin Sensitivity, and Fasting Blood Glucose

When the oral glucose tolerance test was performed at the end of the experimental period (Table 5), a higher glycemic level was observed in all experimental groups that received the extracts at all times. At 30 and 60 min, the hyperlipidemic control group differed significantly (*p* ≤ 0.001) from the DN control group.

In the insulin sensitivity test (Table 6), it was observed that at time zero, the EE50 group presented significantly higher values than the DC, DN, DH, and EE25 groups. Furthermore, the EA25 group differed from the DC, DN, and DH groups, while the EA50 group only showed a difference from DC (*p* ≤ 0.001). There were no differences between the groups at 15 min (*p* = 0.128). At 30 min, the DN control group showed greater insulin resistance when compared to DC (*p* = 0.015). At 60 min, the EE25 and EA25 groups obtained significantly higher values when compared to the DC group (*p* = 0.005).

**Figure 2:**
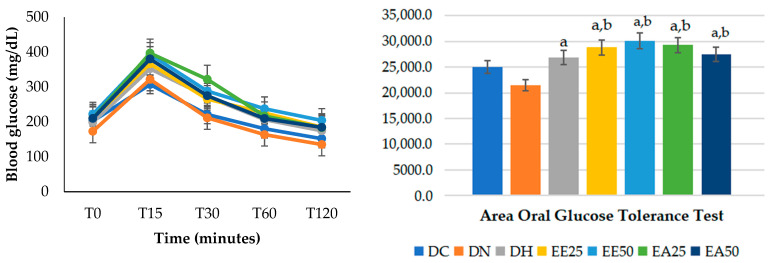
Glucose tolerance test and the area of the glycemic curve. ANOVA followed by Tukey’s post hoc test (*p* ≤ 0.001). Letters indicates statistically significant differences as follows: a indicates a difference with respect to DN; b indicates a difference with respect to DC. DC = commercial diet; DH = high-fat diet; DN = normocaloric diet; EE25 = ethanolic extract at 25 mg/kg; EE50 = ethanolic extract at 50 mg/kg; EA25 = aqueous extract at 25 mg/kg; EA50 = aqueous extract at 50 mg/kg.

When calculating the area under the curve for the oral glucose tolerance test (Figure 2), it was found that all groups supplemented with the aqueous and ethanolic extracts, regardless of the dose, presented statistically significantly higher values when compared to the control groups DC and DN. Furthermore, the HD group differed from the DN group (*p* ≤ 0.001).

**Figure 3:**
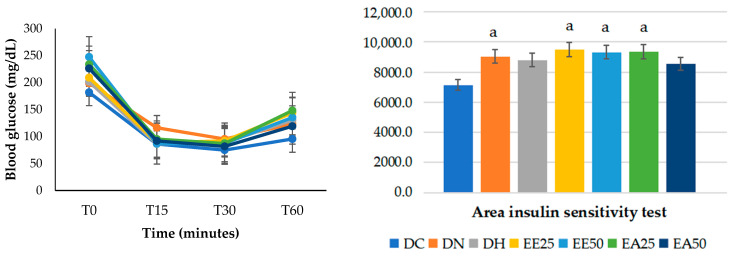
Insulin sensitivity test and glycemic curve area (%). ANOVA followed by Tukey’s post hoc test (*p* ≤ 0.001). Letters indicate statistically significant differences as follows: a indicates a difference with respect to DC. DC = commercial diet; DH = high-fat diet; DN = normocaloric diet; EE25 = ethanolic extract at 25 mg/kg; EE50 = ethanolic extract at 50 mg/kg; EA25 = aqueous extract at 25 mg/kg; EA50 = aqueous extract at 50 mg/kg.

The results of the area under the curve for the insulin sensitivity test (Figure 3) demonstrated statistically significantly higher values (*p* = 0.008) in the DN, EE25, EE50, and EA25 groups when compared with the DC group. In other words, in the model studied, the administration of ethanolic and aqueous extracts did not reduce the animals’ glycemia.

**Figure 4:**
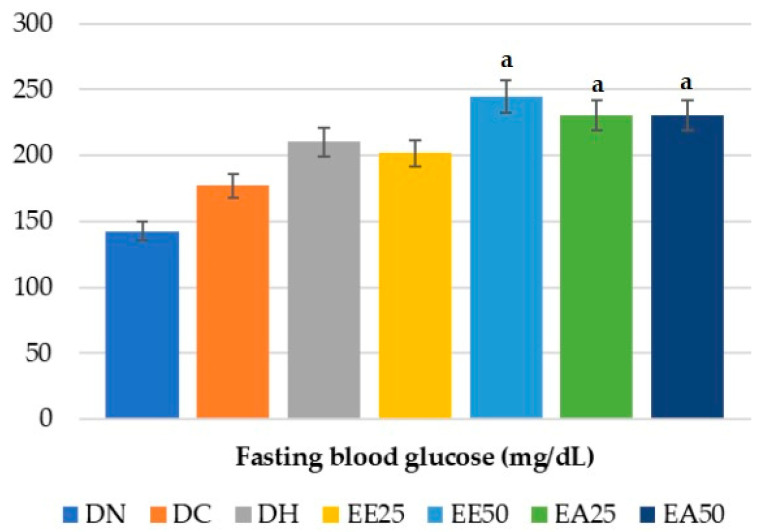
Fasting blood glucose test. ANOVA followed by Tukey’s post hoc test (*p* = 0.006). Letters indicate statistically significant differences as follows: a indicates a difference with respect to DN. DC = commercial diet; DH = high-fat diet; DN = normocaloric diet; EE25 = ethanolic extract at 25 mg/kg; EE50 = ethanolic extract at 50 mg/kg; EA25 = aqueous extract at 25 mg/kg; EA50 = aqueous extract at 50 mg/kg.

Regarding fasting glycemia, the experimental groups EE50, EA25, and EA50 showed statistically significantly higher values when compared to the DN group (*p* = 0.006) (Figure 4).

### 2.4. Serum Analyses

In relation to lipid profile, it was observed that the total cholesterol was higher in the experimental groups that received the extracts than in the control groups (DC and DN) (*p* ≤ 0.001), but there were no differences with respect to DH. HDL-c was higher in the EE25 group than in the DC, DN, EE50, and EA50 groups, and the EA25, EA50, and EE50 groups presented values higher than that of the DC group (*p* ≤ 0.001). Non-HDL in the EE50 and EA50 groups was higher than that in the other groups (*p* ≤ 0.001). LDL-c was higher in EA50 than in the control groups, including the lipid group; the EE50 group differed from DN and DC, and EA25 differed from DN. On the other hand, VLDL-c and triglycerides were lower in the EE25, EE50, and EA50 groups than in the DN group. When the atherogenic index was evaluated, the EE50, EA50, and DC groups obtained significantly higher values compared to the DH, DN, and EE25 groups (*p* ≤ 0.001) (Table 7). Thus, the administration of the ethanolic extract at the lowest dose especially contributed to high HDL-c levels, but neither of the extracts had an influence on the other serum parameters evaluated.

### 2.5. Hormonal and Inflammatory Profile

There was an increase in insulin in the EE50 and EA50 groups in relation to the non-lipid control groups (*p* ≤ 0.001). For leptin, DH, EA25, and EA50 presented significantly higher values than did DC and DN, and the EE25 and EE50 groups differed only from DC (*p* ≤ 0.001). High values for resistin were observed in DH, EA25, and EA50 when compared to DC and DN (*p* ≤ 0.001).

With respect to inflammatory parameters, the EA50 group presented higher values of IL-6 compared to the other groups with a significant difference from DC and EE25 (*p* = 0.005). There were low concentrations of MCP-1 in EA25 when compared with the DH group (*p* = 0.024). It should be noted that all of the groups that received a high-fat diet and some treatment with the extract (in both doses) remained similar to the groups that received a control diet (commercial diet and normocaloric diet), and there were no statistically significant differences between the groups in the concentrations of PAI-1 and TNF-α (Table 8). Thus, the ethanolic and aqueous extracts increased the leptin levels, which may have contributed to satiety, but they were not shown to provide protection through other inflammatory and hormonal parameters.

### 2.6. Histology of the Liver and Pancreas

**Figure 5:**
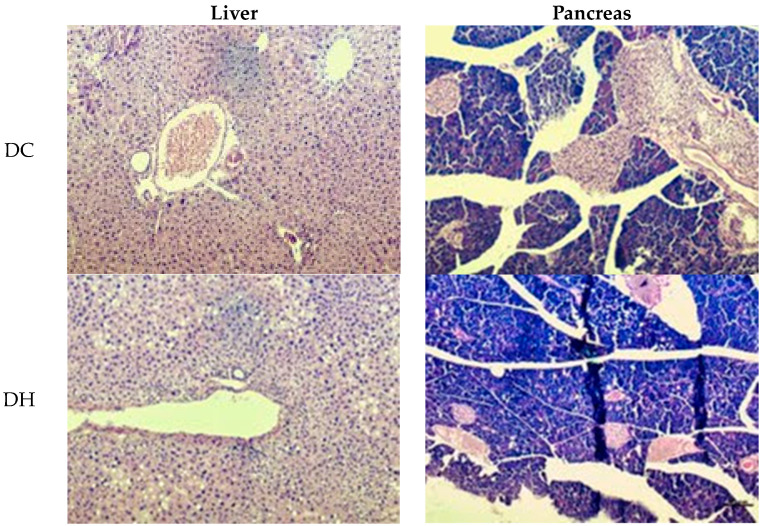

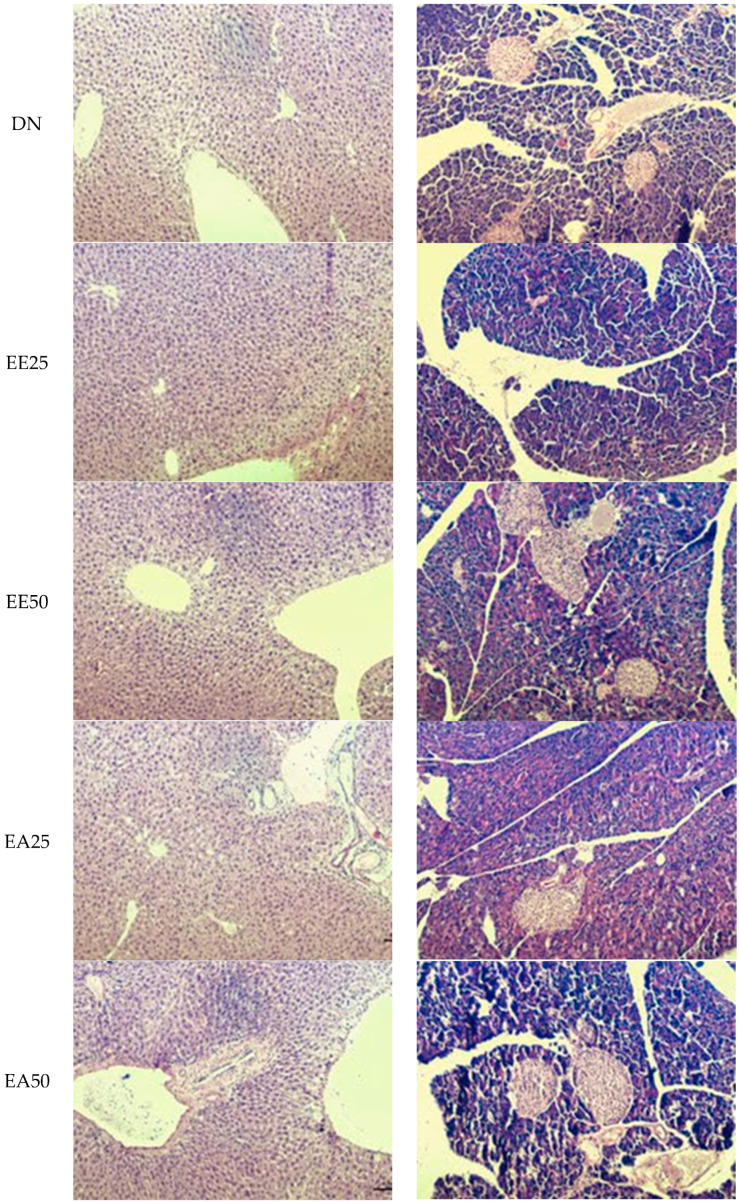
Histological analysis of the liver and pancreas of animals from each of the experimental groups; 100× magnification. DC = commercial diet; DH = high-fat diet; DN = normocaloric diet; EE25 = ethanolic extract at 25 mg/kg; EE50 = ethanolic extract at 50 mg/kg; EA25 = aqueous extract at 25 mg/kg; EA50 = aqueous extract at 50 mg/kg.

The quantification of steatosis, microvesicular steatosis, lobular inflammation, ballooning, Mallory hyaline, apoptosis, and glycogenated nuclei is presented in Table 9 and Figure 5. In the analysis related to ballooning, a difference was observed between the groups (*p* = 0.007), as the EE50 group showed a higher prevalence of a few cells than did the DC and DN groups, without differing from the other groups, just as the other groups did not differ from each other. In the other parameters evaluated in both the liver and pancreas, there were no statistical differences when comparing the groups (*p* > 0.05).

## 3. Discussion

*G. ulmifolia* has been popularly used as a medicinal plant, and in ethnobotanical studies of the leaves, fruit, root, and stem bark, properties such as antidysenteric, antibacterial, anti-inflammatory, antimicrobial, antifungal, depurative, and hepatoprotective activities have been reported. In pharmacological evaluations, antioxidant, antihypertensive, vasodilatory, antidiabetic, antiviral, antibacterial, antifungal, gastroprotective, hepatoprotective, and cytotoxic activities were demonstrated [21,22].

These medicinal properties are associated with the presence of alkaloids, tannins, saponins, flavonoids, terpenoids, glycosides, and steroids, as well as octacosanol, taraxeroloac, friedelin-3-áoac, â-sitosterol, friedelinol-3-acetate, kaempferol, epicatechin oligomers, and procyanidins, such as procyanidin B2, procyanidin B5, and procyanidin C1, in different parts of *G. ulmifolia* [7,23].

In this study, when the chemical composition of the ethanolic and aqueous extracts of *G. ulmifolia* bark was evaluated, it was observed that the secondary metabolites identified had higher concentrations of tannins, which are followed by phenolic compounds and flavonoids. In addition to these, coumarins, triterpenes, steroids, cyanogenic heterosides, cardioactive heterosides, and saponins were also identified. The phytochemical results of this study also showed that the aqueous extract of the stem bark of *G. ulmifolia* had greater antioxidant capacity since it presented lower IC_50_ values [11].

In the extracts, a group of polyphenolics was identified, which includes catechin and its gallic acid conjugates, gallocatechin, epigallocatechin, epigallocatechin gallate, epicatechin, gallocatechin gallate, epicatechin gallate and catechin gallate. The basic functions of this group of catechins include their antioxidant effects: the scavenging of reactive oxygen species (ROS), inhibition of the formation of free radicals and lipid peroxidation [24]. 

The antioxidant efficacy of catechins is exerted through direct mechanisms—scavenging ROS, chelating metal ions, and indirect mechanisms—inducing antioxidant enzymes, inhibiting pro-oxidant enzymes, and producing phase II detoxification enzymes and antioxidant enzymes [25]. Catechin and its diastereoisomers all have common chemical structures, and the number and arrangement of hydroxyl groups is related to their antioxidant potential, since the phenolic hydroxyl group of catechins donate one electron of phenoic OH group, thus reducing free radicals [26]. 

Another compound identified in ethanolic and aqueous extracts was caffeine, which may in a dose-dependent manner play a role in lipid metabolism, since the inhibition of phosphodiesterases exerted by caffeine promotes the accumulation of cyclic adenosine monophosphate (AMPc); consequently, the high concentration of AMPc may promote lipolysis, which involves the hydrolysis of triglycerides into glycerol and free fatty acids in adipose tissue [27,28]. 

Although ethanolic and aqueous extracts from the bark of the stem of *G. ulmifolia* have phytochemical qualities that guarantee their protective and health-promoting effects, it is important to investigate whether they can be toxic. The acute toxicity tests in the present study proved that neither extract interfered with water intake, body weight, the weights of vital organs (kidney, liver, heart, lung, and spleen), or behavioral parameters (“Hippocratic screening”) when offered in low doses in accordance with the OECD [29].

In view of this, in association with the results found in other studies, the composition of the ethanolic and aqueous extracts of the stem bark of *G. ulmifolia* presents relevant nutrients and bioactive compounds, such as antioxidants, with nutraceutical importance and potential health effects [30,31]. However, to date, there are still no studies that prove these properties, nor is there any evidence of the possible beneficial effect of *G. ulmifolia* under conditions of obesity and/or metabolic changes resulting from weight gain and adiposity.

It is known that changing caloric intake triggers possible weight changes and favors the development of metabolic changes, such as diabetes, hypertension, and dyslipidemia [32]. Furthermore, the composition of a diet can influence food consumption, as diets with greater amounts of fat tend to promote satiety for longer, reducing the amount consumed [33].

At the end of the experiment, lower body weight was observed in the EE25 and EA50 groups (without significant differences in relation to the control groups), and this was accompanied by a significant reduction in food and caloric intake in these groups when compared with the DH group [34]. We speculate that a longer supply of the extracts at these doses could reveal a change in body weight with a significant reduction in body weight. It is also recommended that further studies with different doses of the extract be carried out in order to verify the behavior of body weight in relation to food consumption.

On the other hand, there was no impact on adiposity or adipocyte size. In an in vitro study that, unlike this study, used the leaves of *G. ulmifolia*, Nuri et al. [35] revealed that fractions of the ethanolic extract of the leaf have an anti-obesity effect by inhibiting the proliferation and differentiation of pre-adipocytes, which is an effect that was attributed to the presence of a flavonoid—a phenolic compound present in *G. ulmifolia* that modulates lipid metabolism and increases the basal metabolic rate through the modulation of hormone-sensitive lipase, acetyl-coA carboxylase, carnitine acyltransferase, and peroxisome proliferator-activated receptor proteins (PPARs), causing the aforementioned effect [36].

Phenolic compounds are also related to antioxidant effects; it is known that oxidative stress is a condition of imbalance between the number of reactive species and the inefficient activity of an organism’s antioxidant protection system [37], and it is often associated with symptoms and diseases, including diabetes [38]. The antioxidant activity of *G. ulmifolia* extracts against oxidative stress can be partially attributed to the presence of these phenolic compounds because they are capable of chelating metal ions and inhibiting the Fenton reaction—particularly flavonoids such as quercetin and catechin. Furthermore, the presence of aromatic rings allows the donation of H+ and electrons, preventing the formation of ROSs, such as OH + and ROO, which explains the decrease in lipid peroxidation and glycemic control [22]. Thus, combating oxidative stress is a way to control blood glucose, and pharmacological studies have confirmed this antidiabetic potential of the bark of the stem and leaves of *G. ulmifolia* [12,13].

Another way to control glycemia would be to prevent the hydrolysis and absorption of carbohydrates after food intake by inhibiting α-glucosidases that are located in the intestine, retracting the intestinal absorption of carbohydrates, and consequently reducing postprandial glycemia [39]. A study by Contreras et al. [40] suggested that the antidiabetic activity of *G. ulmifolia* would be mediated by the inhibition of α-glucosidase, which, in addition to the digestion process, influences the uptake of carbohydrates after food ingestion, thus controlling the transport of glucose in the blood. The inhibition of α-glucosidase enzymes and prevention of oxidative stress in postprandial hyperglycemia are possible mechanisms by which antidiabetic properties are exerted, which would imply potential for reducing postprandial glucose. However, in the present study, this effect was not confirmed, as lower values of TTOG and TSI were not observed in the groups that received the aqueous and ethanolic extracts from the bark of the stem of *G. ulmifolia,* regardless of the dose, in fasting glycemia; this condition may have been associated with the dosage used in the studies (varying between 1 and 70 µg/mL) and the study format (in vitro), which, unlike the present study, was in vivo.

Additionally related to the composition of bioactive compounds, studies have shown that *G. ulmifolia* can reduce blood cholesterol levels and the occurrence of atherosclerosis, as secondary metabolites—alkaloids, tannins, saponins, flavonoids, terpenoids, and steroids—can reduce markers of inflammation and platelet aggregation and protect against thrombogenesis and oxidative stress in addition to preventing hypercholesterolemia and hypertriglyceridemia [22,30].

In a study carried out by Ramadhansya and collaborators [41], Wistar rats were fed a high-fat diet supplemented with *G. ulmifolia* leaf extract at doses of 0.2, 0.4, and 0.8 g/kg of body weight once per day for 56 days, and the medication orlistat was used at a dose of 2.1 mg three times per day in a control group. It was observed that *G. ulmifolia* improved the lipid profile in such a way that it reduced the development of coronary atherosclerosis and shared a similarity with the therapeutic effect of orlistat.

The results obtained in this study indicated varied changes in the lipid profile, though the groups that received extracts from the bark of the stem of *G. ulmifolia* (with both doses) obtained high values of total cholesterol. However, the lower dose of ethanolic extract contributed to a reduction in VLDL-c, LDL-c, and atherogenic index levels, in addition to an increase in HDL-c values, corroborating the results of the study by Mahfudh and collaborators [42], who verified the effect of *G. ulmifolia* extract in rats fed a high-fat diet, showing that it plays an essential role in anti-hyperlipidemic and hepatoprotective activities.

*G. ulmifolia* has large amounts of saponins, which are related to the inhibition of the activity of pancreatic lipase enzymes and, thus, reduce the absorption of fat in the intestine [43].

The species *G. ulmifolia* contains significant amounts of flavonoids and phenolic compounds [31,44]. Furthermore, regarding the composition of the stem bark of *G. ulmifolia*, the presence of proanthocyanidins is crucial for preserving endogenous antioxidant enzymes, such as superoxide dismutase (SOD), eliminating free radicals through their antioxidant activity (phenolic compounds), inhibiting the release of pro-oxidant agents (antioxidants), and acting with anti-inflammatory action. Studies such as those by Maldini and collaborators [45] and Syaefudin and collaborators [46] showed the anti-inflammatory action of the stem bark of *G. ulmifolia*.

A condition that contrasted with the present study, in which the presence of flavonoids and proanthocyanidins, among other bioactive compounds, did not make it possible to combat the effects of ingesting a high-fat diet, was mainly related to inflammatory factors. The ethanolic and aqueous extracts increased leptin levels, which may have contributed to satiety but was shown to provide no protection in terms of other inflammatory and hormonal parameters. Furthermore, it may have had an influence on the high concentration of adipocytokines found (TNF-α, IL-6, MCP-1, and PAI-1), indicating that under the conditions of this study, even with the presence of bioactive compounds, such as flavonoids, the extracts did not promote anti-inflammatory protection. However, it is worth noting that all of the groups that received a high-fat diet and some treatment with the extract (with both doses) maintained MCP1 values similar to those of the groups that received a commercial diet and a normocaloric diet.

Leptin is a hormone that is directly related to energy metabolism, controlling food intake [47]; it is derived from adipose tissue, suggesting that it is an endocrine gland. Adipose tissue itself is an important target of leptin action, via autocrine, paracrine or endocrine signaling, which are fundamental to its main role in energy homeostasis [48]. This action may differ depending on the depot and the type of adipocyte, white or brown, and includes, in addition to the control of the main pathways of lipid metabolism, other physiological processes such as adipogenesis, apoptosis, thermogenesis and browning, and inflammation.

The blood–brain barrier (BBB) is made up of several highly specialized cell types that protect the brain from toxic substances and regulate the passage of macromolecules as well as the bidirectional transport of nutrients and hormones between the blood and brain. Food intake and metabolism are regulated by different hormones, such as leptin, whose circulating levels must be regulated very precisely and are often altered in obesity. These hormones must reach the brain by crossing the BBB through a specific transporter [49]. As many of these transporters are affected by saturation mechanisms, the circulating levels of hormones affect their activity and regulation, and therefore, transporters at the level of the BBB play a critical role in the regulation of metabolism. Some studies carried out on rodents have shown that feeding a high-fat diet (HFD) produces neuronal loss in the arcuate nucleus and hypothalamus as well as causing a decrease in the integrity of the BBB due to the loss of tanycytes (specialized ependymal cells in the middle eminence) and transporters at the level of the BBB [50].

The adequate regulation of lipid metabolism is essential to prevent the development of obesity and metabolic diseases. Leptin is involved in the regulation of lipid metabolism in the adipose organ, both directly through its interaction with its receptor [51] and indirectly through sympathetic innervation [52]. However, the direct effects of leptin on adipose tissue appear to be modest compared with its actions via the sympathetic nervous system (SNS) [53].

When there are changes in the amount of adipose tissue, as in obesity, large amounts of this hormone are produced; it then loses its action in the central nervous system, thus losing its ability to intervene in food consumption. In addition, leptin acts as an energy regulator in the brain used to induce anorexic factors and suppress appetite factors, reducing intake and increasing energy expenditure [48,51]. However, low levels of leptin can increase food absorption and suppress energy expenditure; in contrast, increased levels of leptin can suppress appetite and increase energy intake [52].

It also has pro-inflammatory activity, that is, its exacerbated presence causes an increase in the expression of TNF-α and IL-6 by monocytes and macrophages [54,55]. In this sense, the results of this study show that higher levels of leptin contribute to a consequent increase in the levels of TNF-α and IL-6, which was observed especially in the group supplemented with the aqueous extract at the highest dose.

In turn, pro-inflammatory cytokines (TNF-α and IL-6) have the ability to induce the expression of resistin, which is directly related to insulin resistance and is associated with the activation of inflammatory processes through a pathway dependent on nuclear factor κB (NF-κB). Thus, the consumption of a high-fat diet intensifies the inflammatory process caused by increased cytokine concentrations and insulin resistance, which will favor the emergence of associated comorbidities [56,57].

The histological evaluation showed that the extracts of *G. ulmifolia* did not have a significant impact on the cellular architecture of the liver and pancreas. In the analysis of the liver, the presence of ballooning was observed. This is a morphological alteration that denotes cellular damage and is common in situations of diets with high levels of carbohydrates and/or lipids as well as constant alterations in blood glucose values that alter the transport and storage of triglycerides in the liver, which is not necessarily a pathological condition [58].

## 4. Materials and Methods

### 4.1. Obtaining Raw Materials

The stem bark of the species *Guazuma ulmifolia* Lam. was collected in the city of Campo Grande, Mato Grosso do Sul, Brazil (georeferencing: 20°30′20″ S and 54°36′28″ W), and an exsiccate was deposited in the CGMS-UFMS Herbarium (n° 29217). The plant was registered with the number A26D547 in the National System for the Administration of Genetic Heritage and Associated Traditional Knowledge (Sisgen).

### 4.2. Preparation of the Ethanolic and Aqueous Extract of the Stem Bark of G. ulmifolia

To obtain the ethanolic extract, the stem peels were dried in an oven with air circulation for 12 h at a maximum temperature of 40 °C. The dried plant material was crushed in a four-knife mill until a fine powder was obtained. Ethanol extraction was carried out with 100 mL of ethanol–water solution (80:20 *v*/*v)*, to which 5 g of fine powder from the stem bark was added. The extraction took place in an ultrasound bath for 30 min. The plant material with ethanol was filtered using filter paper so that the residue could be subjected to two more extractions under the same conditions. The obtained extract was then concentrated in a rotary evaporator at 40 °C and completely dried in a fume hood. The dry ethanolic extract was stored at −18 °C in an amber bottle until use.

To obtain the aqueous extract, 5 g of fine powder from the stem bark was added to 100 mL of water heated to 80 °C, and mechanical stirring was maintained for 20 min on a heating plate. Soon after, it was filtered with the aid of gauze, and the residue was subjected to two more extractions using the same procedure. Subsequently, the material that was obtained was lyophilized until a dry powder was obtained, which was stored in an amber bottle at a temperature of −18 °C until use [59].

### 4.3. Phytochemical Analysis of the G. ulmifolia Stem Bark Extracts

The extracts were subjected to phytochemical prospecting according to the methodology of Matos [60]. To confirm the classes of secondary metabolites, the procedure of Wagner and Bladt [61] was used. To confirm the presence of triterpenes and steroids, the dry methanolic extract was hydrolyzed with potassium hydroxide (0.5 mol/L) and refluxed for 1 h. The extracts were extracted with ethyl ether and subsequently subjected to the Liebermann–Burchard reaction. The characterization analyses assessed the following: phenolic compounds (precipitation reaction with ferric chloride), naphthoquinone (acid/base reaction), flavonoids (cyanidin and sulfuric acid reaction), tannins (reaction with iron salts, protein precipitation), coumarins (KOH/ultraviolet light), triterpenes and steroids (Liebermann–Burchard reaction), cyanogenic heterosides (Guignard test), cardioactive heterosides (Bal-jet and Kedde test), alkaloids (Draggendorf), and saponins (Lieberman–Burchard reaction and the surface action test). The following tests were used: the Liebermann–Burchard reaction (steroid nucleus reaction), Keller–Killiani and Pesez reactions (deoxysugars), Baljet and Kedde reactions (lactone ring), and sugar reduction (Benedict reaction). Analyses were performed in triplicate. To determine the presence of classes of secondary metabolites, the intensities of the characterization reactions were classified as follows: 0 (zero) for a negative reaction (-), partial intensity (+ = 10%), low (++ = 50%), medium (+++ = 75%), and high intensity (++++ = 100%).

#### 4.3.1. Quantification of Phenolic Compounds, Flavonoids, and Tannins

The extracts were solubilized at a concentration of 1 mg mL^−1^ in methanol to carry out analyses of phenolic compounds, flavonoids, and tannins. The content of phenolic compounds was determined based on the Folin–Ciocalteau colorimetric method [62]. To this end, 1.5 mL of 20% aqueous sodium carbonate solution, 0.5 mL of Folin–Ciocalteau reagent (1:10 *v*/*v*), and 1 mL of distilled water were added to 100 µL of each sample. After 30 min of reaction, a spectrophotometer reading was taken at a wavelength of 760 nm. To calculate the concentration, an analytical curve was constructed using gallic acid as a standard at concentrations of 100–1000 µg mL^−1^ (a = 0.0008; b = 0.0015; R^2^ = 0.9875). The result was expressed as mg of gallic acid per g of extract.

The determination of flavonoids followed the methodology proposed by Djeridane et al. [62]. A total of 1000 µL of 2% aluminum chloride (AlCl_3_·6H_2_O) in methanol was added for each 1000 µL of each sample with 15 min of reaction. The reading was carried out on a spectrophotometer at a wavelength of 430 nm. To calculate the concentration of flavonoids, an analytical curve was prepared using rutin as a standard with concentrations of 10–50 µg mL^−1^ (a = 0.0019; b = 0.0105; R^2^ = 0.9990). The result was expressed as mg of rutin per g of extract.

The tannin content was determined using the Folin–Denis spectrophotometric method with tannic acid as a reference [63]. For each 1 mL of sample, 1 mL of Folin–Denis reagent was added, followed by 1 mL of 8% sodium carbonate; this was left to react for 2 h. The reading was carried out on a spectrophotometer at a wavelength of 725 nm. A standard curve of tannin acid ranging from 150 to 400 µg mL^−1^ (a = 0.6983; b = 0.0014; R^2^ = 0.9458) was prepared, and results were expressed in milligrams of tannic acid equivalent (ATE) per gram of extract.

#### 4.3.2. Antioxidant Potential

Dilutions of the samples between 50 and 1000 µg mL^−1^ were made in distilled water in relation to the 2000 µg mL^−1^ preparation and a concentration of 3.24 to 64.52 µg mL^−1^ after the dilution effect of the method. Then, 3 mL of DPPH (1,1-diphenyl-2-picrylhydrazyl) 0.004% in methanol was added to 100 µL of each sample dilution, and the reaction was allowed to take place for 30 min under light and at a controlled temperature (25 °C). After this period, the absorbance of the samples was read using a spectrophotometer at a wavelength of 517 nm [64]. A curve was plotted with the concentrations and percentages of inhibitions, and a straight-line equation was used to obtain the concentration necessary to inhibit 50% of the DPPH radicals (IC_50_). Analyses were performed in triplicate.

#### 4.3.3. Identification of Compounds

A solution at a concentration of 1 mg mL^−1^ was prepared in water. The analyses were performed on a 20A Prominence liquid chromatograph (Shimadzu Co., Kyoto, Japan), which consisted of a degasser, binary pumps, a sample injector, and a diode array detector (DAD). The analysis was performed on a Shim-pack XR-ODS column (2.0 × 75 mm, 2.2 μm) (Shimadzu Co, Japan).

The mobile phase consisted of 0.1% aqueous formic acid (A) and acetonitrile (B) in a gradient elution at a flow rate of 0.3 mL min^−1^. The elution gradient used for separation was as follows: initially 25%; after 2 min, the percentage of B increased to 34% in 7 min, increased to 42% in 9.1 min all at once, and increased to 45% in 30 min. The equilibration time before the next run was 6 min. The flow rate was 0.3 mL min^−1^, the column temperature remained at 40 °C, and the injection volume was 3 μL. The chromatograph was coupled with a quadrupole time-of-flight mass spectrometer (micrOTOF-Q™, Bruker Daltonik GmbH, Bremen, Germany) with electrospray ionization (ESI). The negative mode was used, and spectra were acquired in the mass range of *m*/*z* 50 to 1200. The following were the fitted values for the ESI-MS parameters: a capillary voltage of 4.5 kV; a drying gas temperature of 200 °C; a drying gas flow rate of 9.0 L min^−1^; a nebulizer pressure of 4 bar; collision energy values of 5 and 10 V, with nitrogen as a collision gas. MS data were processed using the Data Analysis 4.4 software (Bruker Daltonics, Bremen, Germany). The identification of compounds of interest was carried out through comparison with the retention time and molecular mass of the respective standard. The quantification of the compounds was carried out through external standardization, and the limits of detection and quantification were determined in extract. Analyses were performed in triplicate.

### 4.4. Experimental Protocol

#### 4.4.1. Acute Toxicity Test

All procedures performed with the animals in this study were submitted to and approved by the Ethics Committee on the Use of Animals of the Federal University of Mato Grosso do Sul under protocol number 1.199/2021.

Female C57BL/6 mice were divided into 3 groups (n = 5). The control group received distilled water (1 mL/kg), and the treatment groups received ethanolic extract and aqueous extract of the stem bark of *G. ulmifolia* at a dose of 2000 mg/kg with the same final volume for both groups; this was administered as a single dose. After treatment, the animals were observed at 30, 60, 120, 240, and 360 min and then daily for 14 days. The presence of cognitive, neuromuscular, and physical alterations was observed, assigning a score of zero to animals that were normal and a grade from 1 to 4 according to the intensity of the changes observed (Hippocratic screening) [20] together with a daily assessment of body weight and food and water consumption. After the toxicity test, the experiment was started [29].

#### 4.4.2. Experimental Design

To carry out this study, 12-week-old male C57BL/6 mice were used; they were provided by the Central Vivarium/UFMS and maintained at a temperature of around 22 ± 2 °C with a 12 h light–dark cycle.

**Figure 6:**
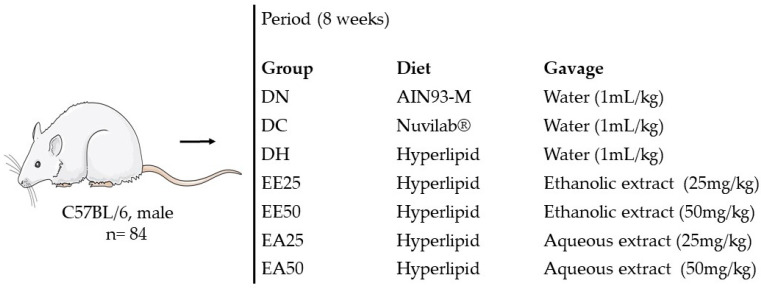
Experimental design.

The mice were divided in groups according to their weight (to initially obtain homogeneous groups according to this parameter), with each group consisting of 12 animals, resulting in a total of 84 animals. The animals were weighed weekly to adjust the doses of extracts and water administered daily in the experimental and control groups, respectively. The distribution of the groups varied according to the food offered and treatment performed. Thus, seven groups were formed as follows: normocaloric diet and distilled water (DN), commercial diet and distilled water (DC), high-fat diet and distilled water (DH), high-fat diet and ethanolic extract from the bark of the stem of *G. ulmifolia* at a concentration of 25 mg/kg (EE25), high-fat diet and ethanolic extract of *G. ulmifolia* stem bark at a concentration of 50 mg/kg (EE50), high-fat diet and aqueous extract of *G. ulmifolia* stem bark at a concentration of 25 mg/kg (EA25), and a high-fat diet and aqueous extract of *G. ulmifolia* stem bark at a concentration of 50 mg/kg (EA50) (Figure 6). Treatments with distilled water (control) and the ethanolic and aqueous extracts of *G. ulmifolia* stem bark were administered via gavage (orally) for 8 weeks [59].

At the end of the experimental period, the animals were initially euthanized using a lethal dose of the inhalational anesthetic isoflurane followed by exsanguination via the inferior vena cava after a 6-hour fast. Blood samples were collected, and the serum was separated through centrifugation for serum analysis.

#### 4.4.3. Diet Composition

During the experimental period, the animals were fed ad libitum with water and a diet for adult mice according to the American Institute of Nutrition (AIN93-M) protocol [34] and the commercial Nuvilab^®^ diet.

The control groups received a commercial Nuvilab^®^ or standard AIN93-M diet, and the other groups received a high-fat AIN93-M diet modified with 31% lard and 20% fructose. Based on the ingredients on its label, the Nuvilab^®^ feed consisted of 60% carbohydrates, 5% lipids, and 22% proteins, while the prepared AIN93-M feed contained 75.8% carbohydrates, 9.5% lipids, and 14.7% proteins, and the hyperlipidic feed contained 31.7% carbohydrates, 57.7% lipids, and 10.6% proteins. The rations were prepared by the company Pragsoluções Biociências.

#### 4.4.4. Control of Body Weight and Food Intake

Mice from all groups were weighed twice per week using a Luxor^®^ digital scale to assess weight gain. Body weight is expressed in grams.

Control of diet intake was monitored twice per week, checking the amount of feed offered to the animals and the remaining amount, expressed in g consumed daily per animal. Energy intake, expressed in kcal/day, was calculated by multiplying the amount of feed consumed by the energy density value of each diet.

The food efficiency coefficient (FEC) was calculated with the purpose of determining how much one gram of food consumed promoted an increase in body weight using the following equation [65]:

$$FEC=\frac{PF-PI}{TA}$$

FEC: food efficiency coefficient;
PF: final body weight in grams;
PI: initial body weight in grams;
TA: total amount of feed consumed in grams.

The calculation of the coefficient of weight gain per caloric intake (CGPCC) was also used with the aim of analyzing the animals’ ability to convert consumed food energy into body weight according to the formula [65]:

CGPCC=(PF−PI)/Kcal consumida

CGPCC: coefficient of weight gain per caloric intake;
PF: final body weight in grams;
PI: initial body weight in grams;
Kcal: caloric value of the diet consumed.

#### 4.4.5. Assessment of Body Fat

After euthanasia, the omental, epididymal, retroperitoneal, perirenal, and mesenteric fat sites of each animal were completely removed and weighed on a semi-analytical scale (Bel^®^) for comparison among the groups. The adiposity index was calculated with the following formula [66]:

% adiposity=sum of visceral white adipose tissue−final body weight×100

### 4.5. Oral Glucose TOLERANCE and Insulin Sensitivity Test

An oral glucose tolerance test was performed 5 days before the animals were euthanized after 6 h of fasting. Firstly, fasting blood glucose was checked via the caudal route (time 0) using a G-Tech^®^ brand glucometer (Worcestershire, UK). Then, the animals received a D-glucose solution at a concentration of 2 g/kg of body weight via gavage. Blood glucose readings were taken 15, 30, 60, and 120 min after applying glucose [67].

Insulin sensitivity testing was performed 3 days before euthanasia. With the animals in a fed state, blood glucose levels were checked (time 0) using the G-Tech^®^ glucometer. Then, 0.75 IU of insulin (Novorapid^®^—100 U/mL) per kg of animal weight was injected intraperitoneally. Blood glucose readings were taken at 15, 30, and 60 min [67].

From the peak blood glucose values, the area under the curve for each group in both the oral glucose tolerance test and the insulin sensitivity test was determined [68].

### 4.6. Serum Biochemical Analyses

Plasma concentrations of triglycerides, cholesterol (total and fractions), HDL cholesterol, and blood glucose were measured according to the guidelines of a commercial kit from Lab Test Diagnóstica^®^, Lagoa Santa, Minas Gerais, Brazil.

The determination of the atherogenic index was carried out by calculating the ratio between total cholesterol and HDL cholesterol [69].

### 4.7. Hormonal and Inflammatory Profile

The blood collected after euthanasia was centrifuged, and the supernatant was stored in a biofreezer at −80 °C. To determine the concentration of adipocytokines present in the serum, the samples were thawed, mixed in a vortex for 30 s and centrifuged at 6000 rpm for 10 min. Next, 10 μL of the serum from each animal was placed in a 96-well plate along with 10 μL of an assay buffer solution and 25 μL of a solution containing seven adipokines. We also prepared the blank, standard and control parameters, following the instructions (Milliplex^®^ MAP kit, Billerica, MA, USA). The adipokines, which include IL-6, MCP-1, TNF-α, PAI-1, insulin, leptin and resistin, were quantified using the commercial MAD-KMAG-71K kit (Merck-Sigma Aldrich, São Paulo, Brazil). The plates were analyzed on the Luminex MAGPIX system (Luminex Corporation, Austin, TX, USA), and the data were generated using the xPONENT 4.3 software. Luminex^®^ software. All procedures for carrying out this analysis were carried out in accordance with the manufacturer’s manual.

### 4.8. Histology of Epididymal Adipose Tissue, Liver, and Pancreas

After removing and weighing the liver, pancreas, and epididymal adipose tissue, fragments of these organs were fixed at 10% until being embedded in paraffin. After fixation, they were cut with a microtome at a thickness of 5 μm each and stained with hematoxylin–eosin [70].

The histological analysis of the liver was performed using the Kleiner system [71], and the architecture of the pancreas was evaluated according to the methodology of Chandran et al. [72].

To analyze the area of adipocytes in epididymal adipose tissue, images were initially captured using the LEICA DFC 495 digital camera system (Leica Microsystems, Wetzlar, Germany), which was integrated into a LEICA DM 5500B microscope (Leica Microsystems, Wetzlar, Germany) with 20× magnification. The images were analyzed using the LEICA Application Suite version 4.0 software (Leica Microsystems, Wetzlar, Germany), and the average area of 100 adipocytes per sample was determined [73].

### 4.9. Statistical Analysis

The results are expressed as the mean ± standard error of the mean. Analysis of variance (ANOVA) was used for multiple comparisons of parametric results, which was followed by Tukey’s post hoc test, and for non-parametric data, the Kruskal–Wallis and Friedman tests were used, which were followed by Dunn’s post hoc test. To carry out the statistical analysis, the Sigma Stat software (version 3.5, Systal Software, Inc., San Jose, CA, USA) was used. The chi-square test was used to evaluate the associations in histological analyses, followed by the Bonferroni correction, using the Bioestat 5.0 statistical program. The significance level adopted was *p* < 0.05.

## 5. Conclusions

The evaluation of the phytochemical composition of the ethanolic and aqueous extracts of the stem bark of *G. ulmifolia* identified compounds similar to those reported in the literature. In an acute toxicity model, the extracts did not promote any neurological or behavioral changes or mortality in the animals, although there was a reduction in food intake in agreement with the subsequent results of the study. In the experimental model, lower food consumption was observed in the EE25 and EA50 groups than in the control groups. The ethanolic and aqueous extracts, regardless of the dose, did not influence weight gain or visceral adiposity, and they caused varied changes in the lipid profile. They also did not contribute to better glycemic levels in the glucose tolerance, insulin sensitivity, and fasting glucose tests.

Furthermore, under the conditions of this study, the ethanolic and aqueous extracts increased leptin levels, which may contribute to satiety, but they were shown to have a negative impact on other inflammatory and hormonal parameters in mice that were fed a high-fat diet. Therefore, it is suggested that studies with different experimental conditions and study times be carried out in order to clarify the empirical use of this plant in the treatment of comorbidities associated with excessive fat consumption; in addition, it is suggested to carry out the isolation of fractions and/or compounds from the extracts that could promote benefits.

## Figures and Tables

**Table 1:** Phytochemical composition and antioxidant activity (DPPH) of ethanolic and aqueous extracts of *Guazuma ulmifolia* stem bark.

Classes	Aqueous Extract	Ethanolic Extract
Phenolic compounds	++	++
Flavonoids	++	++
Tannins	+++	+++
Naphthoquinone	−	−
Coumarins	+	+
Triterpenes and steroids	+	+
Cyanogenic heterosides	+	+
Cardioactive heterosides	+	+
Reducing sugars	+	−
Saponins	+	+
Alkaloids	−	−
TCF [mg g^−1^]	11.1	13.8
TF [mg g^−1^]	3.1	3.3
TT [mg g^−1^]	72.6	65.2
DPPH [µg mL^−1^]	37.3	45.2

Classification of the presence of classes of secondary metabolites and intensities of characterization reactions: 0 (zero) for a negative reaction (−), partial intensity (+ = 10%), low (++ = 50%), and medium (+++ = 75%). TCF = phenolic compound content; TF = flavonoid content; TT = tannin content.

**Table 2:** Compounds identified in ethanolic and aqueous extracts of *Guazuma ulmifolia* stem bark.

Compounds	Correlation Coefficient (r^2^)	LOD (pg/Injection)	LOQ (pg/Injection)	AEB (% m:m)	EEB (% m:m)
Gallocatechin	0.9994	0.15	0.50	5.84 ± 0.17	4.27 ± 0.12
Epigallocatechin	0.9996	0.14	0.47	5.33 ± 0.11	4.54 ± 0.0 6
Catechin	0.9994	0.15	0.50	3.73 ± 0.11	2.87 ± 0.11
Epigallocatechin gallate	0.9992	0.14	0.47	5.99 ± 0.22	5.14 ± 0.11
Epicatechin	0.9992	0.15	0.50	3.29 ± 0.09	2.45 ± 0.07
Gallocatechin gallate	0.9996	0.17	0.44	4. 31 ± 0.11	3. 38 ± 0.13
Caffeine	0.9994	0.12	0.40	4.15 ± 0.07	3.37 ± 0.05
Epicatechin gallate	0.9992	0.18	0.60	2.98 ± 0.06	1.88 ± 0.05
Catechin gallate	0.9990	0.14	0.47	3.05 ± 0.05	1.95 ± 0.08

Calibration curve range (ug mL^−1^) = 20–1000. LOD = limit of detection. LOQ = limit of quantitation. AEB = aqueous bark extract. EEB = ethanolic bark extract.

**Table 3:** Initial and final weight, weight gain, and food intake of animals in each of the experimental groups.

Parameters	Experimental Groups						
	DN	DC	DH	EE25	EE50	EA25	EA50
Starting weight (g)	24.67 ± 1.50	24.75 ± 1.14	24.42 ± 1.56	24.75 ± 1.82	24.17 ± 2.44	24.00 ± 1.60	24.25 ± 1.54
Final weight (g)	29.00 ± 2.00	28.33 ± 0.78	30.25 ± 3.05	29.33 ± 3.23	31.00 ± 3.13	29.58 ± 3.78	29.08 ± 5.28
Total weight gain (g) **	4.33 ± 2.06	3.58 ± 1.17	5.83 ± 2.33	4.58 ± 1.98	6.83 ± 3.01 ^b^	5.58 ± 3.00	4.83 ± 4.09
Total food intake (g) *	50.77 ± 4.19	64.52 ± 3.50	43.69 ± 2.00	37.63 ± 2.08 ^a,b,c^	38.39 ± 3.16 ^a,b^	38.57 ± 3.62 ^a,b^	37.25 ± 3.46 ^a,b,c^
Total caloric intake (kcal) *	192.92 ± 15.91 ^b^	281.31 ± 15.26	231.57 ± 10.62 ^a^	199.45 ± 11.03 ^b,c^	203.49 ± 16.73 ^b^	204.42 ± 19.17 ^b^	197.42 ± 18.36 ^b,c^
CEA *	0.093 ± 0.031	0.056 ± 0.019	0.134 ± 0.053 ^b^	0.121 ± 0.049	0.180 ± 0.086 ^a,b^	0.142 ± 0.070 ^b^	0.124 ± 0.097
CGPCC *	0.027 ± 0.006 ^b^	0.014 ± 0.003	0.025 ± 0.010	0.024 ± 0.008	0.034 ± 0.016 ^b^	0.034 ± 0.011 ^b^	0.028 ± 0.014 ^b^

Values are expressed as the mean ± standard deviation. ANOVA followed by Tukey and Kruskal–Wallis post hoc tests and by Dunn’s test (** *p* = 0.032; * *p* ≤ 0.001). In the same line, a letter indicates a statistically significant difference as follows: a indicates a difference with respect to DN; b indicates a difference with respect to DC; c indicates a difference with respect to DH. DC = commercial diet; DH = high-fat diet; DN = normocaloric diet; EE25 = ethanolic extract at 25 mg/kg; EE50 = ethanolic extract at 50 mg/kg; EA25 = aqueous extract at 25 mg/kg; EA50 = aqueous extract at 50 mg/kg; CEA = food efficiency coefficient; CGPCC = coefficient of weight gain per calorie consumed.

**Table 4:** Weight of adipose tissue sites, percentage of body fat, and adipocyte area of animals in each of the experimental groups.

Groups	Adipose Tissue (mg)					% Adiposity *	Adipocyte Area * (µm^2^)
	Epididymal *	Perirenal *	Retroperitoneal *	Mesenteric	Omental		
DN	348.70 ± 120.84	52.52 ± 22.91	92.50 ± 39.81	199.89 ± 55.74	31.69 ± 13.68	2.50 ± 0.66	2,286,227.97 ± 515,840.80
DC	222.59 ± 84.00	37.98 ± 24.69	44.02 ± 20.27	172.60 ± 59.10	40.08 ± 10.64	1.82 ± 0.54	1,881,104.03 ± 296,416.70
DH	699.05 ± 289.18 ^b^	122.08 ± 67.05 ^b^	284.06 ± 119.99 ^b^	241.34 ± 80.51	45.44 ± 14.62	4.53 ± 1.24 ^b^	3,969,639.00 ± 1,308,460.12 ^a,b^
EE25	814.51 ± 399.18 ^a,b^	121.37 ± 67.05 ^b^	305.86 ± 167.83 ^a,b^	245.97 ± 113.87	41.99 ± 9.25	5.04 ± 1.89 ^a,b^	3,271,761.53 ± 1,178,744.39 ^b^
EE50	864.77 ± 406.58 ^a,b^	109.46 ± 60.49 ^b^	363.60 ± 186.45 ^a,b^	266.17 ± 103.60	43.57 ± 11.44	5.18 ± 1.83 ^a,b^	3,489,449.25 ± 1,054,370.87 ^b^
EA25	866.92 ± 439.16 ^a,b^	102.20 ± 46.66 ^b^	337.65 ± 177.64 ^a,b^	295.90 ± 144.08	45.11 ± 17.64	5.46 ± 2.15 ^a,b^	3,595,736.22 ± 750,844.13 ^a,b^
EA50	782.30 ± 553.90 ^b^	95.67 ± 74.44	279.38 ± 199.22 ^b^	207.51 ± 154.11	41.78 ± 12.15	4.50 ± 2.43 ^b^	3,315,501.00 ± 1,393,916.23 ^b^

Values are expressed as the mean ± standard deviation. In the same column, letters indicate statistical differences as follows: a indicates a difference with respect to DN; b indicates a difference with respect to DC. ANOVA followed by Tukey’s post hoc test (* *p* ≤ 0.001). DC = commercial diet; DH = high-fat diet; DN = normocaloric diet; EE25 = ethanolic extract at 25 mg/kg; EE50 = ethanolic extract at 50 mg/kg; EA25 = aqueous extract at 25 mg/kg; EA50 = aqueous extract at 50 mg/kg; %BF = percentage of body fat.

**Table 5:** Oral glucose tolerance test on animals according to each experimental group at each time evaluated.

Groups	Time (min)				
	0 *	15 **	30 **	60 **	120 **
DC	202.00 ± 21.64	307.00 ± 34.83	221.00 ± 46.40	180.25 ± 29.28	151.75 ± 21.18
DN	172.92 ± 30.68	321.50 ± 45.00	211.33 ± 45.82	163.25 ± 32.80	135.08 ± 40.90
DH	198.83 ± 29.01	352.08 ± 42.79	271.67 ± 64.94 ^b^	206.33 ± 26.74 ^b^	174.50 ± 30.22
EE25	210.67 ± 33.65 ^b^	366.25 ± 34.36 ^a^	265.36 ± 37.04	225.33 ± 41.17 ^a,b^	184.92 ± 30.76 ^b^
EE50	221.92 ± 25.65 ^b^	393.17 ± 36.36 ^a,b^	288.08 ± 30.46 ^a,b^	237.42 ± 36.05 ^a,b^	204.08 ± 31.34 ^a,b^
EA25	209.67 ± 34.99 ^b^	396.67 ± 33.01 ^a,b^	321.83 ± 36.36 ^a,b^	216.58 ± 25.96 ^b^	183.25 ± 29.76 ^b^
EA50	209.67 ± 29.78 ^b^	380.00 ± 52.52 ^a,b^	274.67 ± 54.68 ^b^	209.42 ± 30.87 ^b^	184.17 ± 40.38 ^b^

Values are expressed as the mean ± standard deviation. ANOVA followed by Tukey’s post hoc test (* *p* = 0.007; ** *p* ≤ 0.001). In the same column, a letter indicates a statistical difference as follows: a indicates a difference with respect to DC; b indicates a difference with respect to DN. DC = commercial diet; DH = high-fat diet; DN = normocaloric diet; EE25 = ethanolic extract at 25 mg/kg; EE50 = ethanolic extract at 50 mg/kg; EA25 = aqueous extract at 25 mg/kg; EA50 = aqueous extract at 50 mg/kg.

**Table 6:** Insulin sensitivity test on animals according to each experimental group at each time evaluated.

Groups	Time (min)			
	0 *	15	30 **	60 ***
DC	181.82 ± 13.40	86.12 ± 10.69	74.92 ± 14.91	95.42 ± 31.87
DN	199.00 ± 30.81	116.33 ± 28.51	95.42 ± 15.36 ^a^	123.25 ± 39.45
DH	201.58 ± 18.77	86.18 ± 14.96	89.18 ± 11.66	130.09 ± 17.43
EE25	209.08 ± 16.82	88.75 ± 22.03	92.00 ± 8.89	143.75 ± 28.36 ^a^
EE50	247.42 ± 27.22 ^a,b,c,d^	86.82 ± 18.14	87.33 ± 14.29	135.25 ± 38.93
EA25	233.83 ± 30.29 ^a,b,c^	95.27 ± 40.17	87.09 ± 13.61	148.17 ± 42.61 ^a^
EA50	226.25 ± 24.86 ^a^	92.00 ± 29.12	81.73 ± 7.31	119.18 ± 21.27

Values are expressed as the mean ± standard deviation. ANOVA followed by the Tukey and Kruskal–Wallis post hoc tests and by Dunn’s post hoc test (* *p* ≤ 0.001; ** *p* = 0.015; *** *p* = 0.005). In the same column, a letter indicates a statistical difference as follows: a indicates a difference with respect to DC; b indicates a difference with respect to DN; c indicates a difference with respect to DH; d indicates a difference with respect to EE25. DC = commercial diet; DH = high-fat diet; DN = normocaloric diet; EE25 = ethanolic extract at 25 mg/kg; EE50 = ethanolic extract at 50 mg/kg; EA25 = aqueous extract at 25 mg/kg; EA50 = aqueous extract at 50 mg/kg.

**Table 7:** Lipid profile and fasting blood glucose of animals in each of the experimental groups.

Parameters (mg/dL)	Experimental Groups						
	DN	DC	DH	EE25	EE50	EA25	EA50
Total cholesterol *	174.33 ± 14.39	145.41 ± 17.05	207.92 ± 20.21 ^b^	236.53 ± 28.40 ^a,b^	231.68 ± 34.36 ^a,b^	228.00 ± 27.43 ^a,b^	243.71 ± 41.13 ^a,b,c^
LDL-c *	25.69 ± 9.66	34.73 ± 19.96	41.79 ± 24.66	46.91 ± 13.69	81.44 ± 28.72 ^a,b^	63.64 ± 24.49 ^a^	92.60 ± 29.00 ^a,b,c^
HDL-c *	111.08 ± 8.73	77.33 ± 7.67	138.79 ± 13.84 ^b^	152.74 ± 14.87 ^a,b,e,g^	118.49 ± 17.43 ^b^	134.19 ± 20.55 ^b^	119.20 ± 17.53 ^b^
Non-HDL *	64.29 ± 9.39	63.92 ± 14.81	75.95 ± 24.47	78.51 ± 15.06	113.14 ± 27.58 ^a,b,c,d^	96.01 ± 24.93 ^a,b^	124.51 ± 31.11 ^a,b,c,d,f^
VLDL-c *	37.86 ± 5.00	33.35 ± 4.34	34.16 ± 4.98	31.76 ± 3.73 ^a^	31.73 ± 4.95 ^a^	32.44 ± 4.13	31.91 ± 4.83 ^a^
Triglycerides **	189.29 ± 24.99	166.77 ± 21.72	170.79 ± 24.89	158.78 ± 18.67 ^a^	158.65 ± 24.75 ^a^	162.20 ± 20.67	159.54 ± 24.13 ^a^
Atherogenic index *	1.58 ± 0.08	1.90 ± 0.28 ^a,c,d^	1.55 ± 0.17	1.55 ± 0.15	1.97 ± 0.25 ^a,c,d^	1.74 ± 0.26	1.99 ± 0.17 ^a,c,d^

Values are expressed as the mean ± standard deviation. ANOVA followed by the Tukey and Kruskal–Wallis post hoc tests and by Dunn’s post hoc test (* *p* ≤ 0.001; ** *p* = 0.018). In the same line, a letter indicates a statistical difference as follows: a indicates a difference with respect to DN; b indicates a difference with respect to DC; c indicates a difference with respect to DH; d indicates a difference with respect to EE25; e indicates a difference with respect to EE50; f indicates a difference with respect to EA25; and g indicates a difference with respect to EA50. DC = commercial diet; DH = high-fat diet; DN = normocaloric diet; EE25 = ethanolic extract at 25 mg/kg; EE50 = ethanolic extract at 50 mg/kg; EA25 = aqueous extract at 25 mg/kg; EA50 = aqueous extract at 50 mg/kg.

**Table 8:** Serum values of inflammatory and hormonal markers of animals in each of the experimental groups.

Parameters	Experimental Groups						
	DN	DC	DH	EE25	EE50	EA25	EA50
IL-6 *	7.77 ± 3.92	4.87 ± 1.22	16.12 ± 16.60	5.97 ± 2.84	9.65 ± 5.27	7.89 ± 3.99	36.11 ± 37.16 ^b,c^
MCP-1	12.46 ± 2.04	13.69 ± 3.05	23.62 ± 10.02 ^d^	14.07 ± 5.61	12.45 ± 2.52	12.10 ± 3.33	13.93 ± 3.82
PAI-1	6148.50 ± 2119.77	4494.81 ± 2211.39	6037.90 ± 2034.07	5694.18 ± 2602.76	6515.00 ± 3422.14	7893.30 ± 4047.81	5052.20 ± 1647.79
TNF-α	6.41 ± 1.36	6.74 ± 0.73	7.23 ± 1.62	5.92 ± 0.65	6.44 ± 0.91	6.28 ± 1.00	6.63 ± 1.12
Insulin **	183.87 ± 70.18	251.06 ± 133.25	419.91 ± 233.98	328.92 ± 161.64	464.95 ± 235.57 ^a^	370.23 ± 169.16	910.98 ± 415.21 ^a,b^
Leptin **	974.08 ± 728.57	512.75 ± 394.71	15,019.60 ± 11,375.73 ^a,b^	6145.16 ± 4467.20 ^b^	8797.24 ± 6874.92 ^b^	10,168.60 ± 7534.31 ^a,b^	19,221.45 ± 12,480.90 ^a,b^
Resistin **	834.89 ± 220.99	920.63 ± 205.49	2118.72 ± 1004.43 ^a,b^	1423.19 ± 406.24	1581.25 ± 730.71	2266.64 ± 1247.78 ^a,b^	2412.67 ± 963.52 ^a,b^

Values are expressed as the mean ± standard deviation. Kruskal–Wallis followed by Dunn’s post hoc test (* *p* = 0.005, ** *p* ≤ 0.001). In the same line, a letter indicates a statistical difference as follows: a indicates a difference with respect to DN; b indicates a difference with respect to DC; c indicates a difference with respect to EE25; d indicates a difference with respect to EA25. DC = commercial diet; DH = high-fat diet; DN = normocaloric diet; EE25 = ethanolic extract at 25 mg/kg; EE50 = ethanolic extract at 50 mg/kg; EA25 = aqueous extract at 25 mg/kg; EA50 = aqueous extract at 50 mg/kg.

**Table 9:** Distribution of changes observed in the liver and pancreas of animals in each of the experimental groups.

Parameters Evaluated	DC		DH		DN		EE25		EE50		EA25		EA50	
Changes in the Liver	n	%	n	%	n	%	n	%	n	%	n	%	n	%
Steatosis														
<5%	13	100.0	11	84.6	12	100.0	10	83.3	13	100.0	12	100.0	11	91.7
5 to 33%	-	-	2	15.4	-	-	2	16.7	-	-	-	-	1	8.3
34 to 66%	0	0.0	0	0.0	0	0.0	0	0.0	0	0.0	0	0.0	0	0.0
<66%	0	0.0	0	0.0	0	0.0	0	0.0	0	0.0	0	0.0	0	0.0
Microvesicular steatosis														
Absent	13	100.0	13	100.0	12	100.0	11	91.7	13	100.0	12	100.0	11	91.7
Presence	-	-	-	-	-	-	1	8.3	-	-	-	-	1	8.3
Lobular Inflammation														
Absent	7	53.8	3	23.1	6	50.0	6	50.0	2	15.4	2	16.7	9	75.0
<2 focuses per 200× field	6	46.2	9	69.2	6	50.0	6	50.0	10	76.9	10	83.3	3	25.0
2–4 focuses per 200× field	-		1	7.7	-		-		1	7.7	-		-	
>4 focuses per 200× field	0	0.0	0	0.0	0	0.0	0	0.0	0	0.0	0	0.0	0	0.0
Ballooning *														
Absent	12	92.3	7	53.8	11	91.7	7	58.3	4	30.8	6	50.0	5	41.7
Few cells	1	7.7 ^b^	6	46.2 ^ab^	1	8.3 ^b^	5	41.7 ^ab^	9	86.2 ^a^	6	50.0 ^ab^	7	58.2
Many cells	0	0.0	0	0.0	0	0.0	0	0.0	0	0.0	0	0.0	0	0.0
Mallory’s Hyaline														
Absent	13	100.0	13	100.0	12	100.0	12	100.0	13	100.0	12	100.0	12	100.0
Presence	0	0.0	0	0.0	0	0.0	0	0.0	0	0.0	0	0.0	0	0.0
Apoptosis														
Absent	10	76.9	9	69.2	9	75.0	8	66.7	6	46.2	8	66.7	8	66.7
Presence	3	23.1	4	30.8	3	25.0	4	33.3	7	53.8	4	33.3	4	33.3
Glycogenated core														
None/rare	13	100.0	13	100.0	12	100.0	12	100.0	13	100.0	12	100.0	12	100.0
Some	0	0.0	0	0.0	0	0.0	0	0.0	0	0.0	0	0.0	0	0.0
Changes in the Pancreas														
Islet of Langerhans														
No change	9	86.2	12	92.3	11	91.7	11	91.7	9	86.2	10	83.3	11	91.7
Mild atrophy	0	0.0	0	0.0	0	0.0	0	0.0	0	0.0	0	0.0	0	0.0
Atrophy	4	30.8	1	7.7	1	8.3	1	8.3	4	30.8	2	16.7	1	8.3
Mild hypertrophy	0	0.0	0	0.0	0	0.0	0	0.0	0	0.0	0	0.0	0	0.0
Hypertrophy	0	0.0	0	0.0	0	0.0	0	0.0	0	0.0	0	0.0	0	0.0
Pancreatic acini														
No change	13	100.0	13	100.0	12	100.0	12	100.0	13	100.0	12	100.0	12	100.0
Necrosis/Atrophy	0	0.0	0	0.0	0	0.0	0	0.0	0	0.0	0	0.0	0	0.0
Inflammatory cells														
No change	13	100.0	13	100.0	12	100.0	12	100.0	13	100.0	12	100.0	12	100.0
Insulitis	0	0.0	0	0.0	0	0.0	0	0.0	0	0.0	0	0.0	0	0.0
Perinsulitis	0	0.0	0	0.0	0	0.0	0	0.0	0	0.0	0	0.0	0	0.0

Data are presented as percentages. *p*-values are according to the chi-square test (* *p* = 0.007). Significant associations; letters in the body of the table indicate differences between groups using Bonferroni correction. DC = commercial diet; DH = high-fat diet; DN = normocaloric diet; EE25 = ethanolic extract at 25 mg/kg; EE50 = ethanolic extract at 50 mg/kg; EA25 = aqueous extract at 25 mg/kg; EA50 = aqueous extract at 50 mg/kg.

## Data Availability

The original contributions presented in the study are included in the article, further inquiries can be directed to the corresponding author.
